# The Nature of Word Associations in Sentence Contexts

**DOI:** 10.1027/1618-3169/a000547

**Published:** 2022-06-13

**Authors:** Clara Planchuelo, Francisco Buades-Sitjar, José Antonio Hinojosa, Jon Andoni Duñabeitia

**Affiliations:** ^1^ Centro de Investigación Nebrija en Cognición (CINC), Language and Education School, Nebrija University, Madrid, Spain; ^2^ Instituto Pluridisciplinar, Facultad de Psicología, Universidad Complutense de Madrid, Madrid, Spain; ^3^ Department of Language and Culture, UiT The Arctic University of Norway, Tromsø, Norway

**Keywords:** word association, valence, arousal, concreteness, sentence generation, semantic representations

## Abstract

**Abstract.** How words are interrelated in the human mind is a scientific topic on which there is still no consensus, with different views on how word co-occurrence and semantic relatedness mediate word association. Recent research has shown that lexical associations are strongly predicted by the similarity of those words in terms of valence, arousal, and concreteness ratings. In the current study, we aimed at extending these results to more complex and realistic linguistic scenarios, since human communication is not done with word pairs, but rather through sentences. Hence, the aim of the current study was to verify whether valence, arousal, and concreteness also articulate sentence-level lexical representations. To this end, 32 native Spanish speakers were given cue words and asked to use them in sentences that would provide a meaningful context. The content words of the written sentences were then analyzed. Our results showed that the emotional dimensions (valence and arousal) and concreteness values of the cue words effectively predicted the same values of said dimensions of their sentences’ words. In sum, the similarity in the emotional dimensions and concreteness are crucial mechanisms behind word association in the human mind.


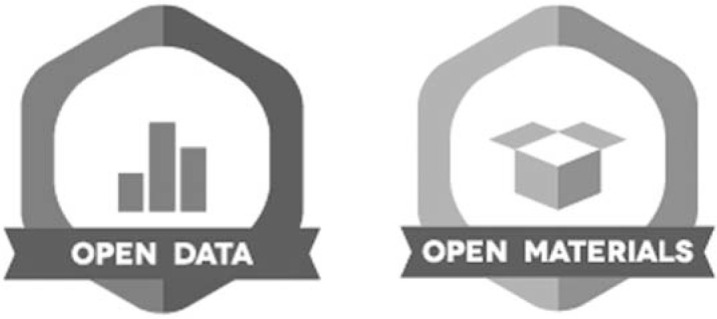


The way in which words are represented and organized in the mental lexicon has always been a topic of great interest for psycholinguists. Language greatly shapes the way we think and how we process the world around us (Barrett et al., 2007; Chen et al., 2014; Lupyan et al. 2020). Understanding how words are encoded in our mental lexicon and how each of them is interrelated to the others is critical to fully comprehend the human mind. As such, it comes as no surprise that several theories have arisen trying to explain how words are represented in the mind, such as the dual coding theory (Paivio, 1971), the blending theory (Coulson & Oakley, 2001), and, more recently, neural network models (Mikolov & Zweig, 2012). Similarly, countless of methodological paradigms have also been designed to validate, refute, and modify these theories, such as lexical decision (e.g., Kuperman et al., 2014; Scott et al., 2014), feature listing (e.g., Chaigneau et al., 2018), and affective priming (e.g., Rossell & Nobre, 2004; Sass et al., 2012).

A relatively unresearched paradigm is that of word association. In a typical word association task, participants are given a cue word and are asked to respond to it with the first word that comes to mind – known as the *associate* – (De Deyne & Storms, 2015). Originally, the paradigm was utilized to detect behavioral and cognitive abnormalities (Jung, 1910; Kent & Rosanoff, 1910) and later as a measure of second language proficiency (Kruse et al., 1987). However, nowadays, word association is mainly used as an exploratory tool to study how words are represented in the human lexicon. The fact that a cue word devoid of any context is able to evoke another word can provide us with valuable clues on how words are organized. Hence, some researchers have invested plenty of resources into creating extensive word association databases (e.g., De Deyne et al., 2019; Fernández et al., 2018), registering the associates given by large numbers of participants in response to tens of thousands of cues. Such large sample and item sizes make these databases an outstandingly reliable source of information on word association for psycholinguists.

Word association and the factors determining the strength of the association between two words (namely, how often a given cue elicits a specific associate) have a huge influence on word processing at multiple levels. For instance, Steyvers et al. (2005) found that, when attempting to recall a list of words, participants' commission errors – that is, indicating words that were not present in the list they had to remember – tended to correspond with words that were strongly associated with words they had to recall (e.g., *dream*-*sleep*). Similarly, when participants were cued with a word that was strongly associated with one or more words from the list, they were much better able to remember said words than those that were not associated with the cue. Another example are studies on cued lexical decision, which have shown that words are more quickly recognized if they are preceded by a strong associate (Lucas, 2000). A different line of research has focused not only on the strength of individual cue–associate pairs but rather on the number of associates one specific cue word is able to elicit and its influence in word processing (e.g., Duñabeitia et al., 2008). Words with a larger number of associates are recognized, named, and read faster than words with a smaller associative neighborhood. Finally, one last line of research has shown that word association seems to be an underlying mechanism behind the differential processing of certain kinds of words, particularly concrete and abstract words (i.e., words that do vs. do not represent concepts with a material basis in the world). Crutch and Warrington (2005) and Crutch and Jackson (2011) found that it took participants more time to find a concrete word embedded in an array of semantically similar words (e.g., *rock* presented along with *boulder* and *pebble*) compared to when embedded in an array of word associates (e.g., *rock* along with *music* and *paper*). Interestingly, when the target was an abstract word, the inverse pattern was found, and it took participants longer to find an abstract word embedded in an array of word associates (e.g., *holy* along with *water* and *spirit* took longer than along with *sacred* and *divine*). The authors suggested that a different organizational system for concrete and abstract words exists in the human mind, with concrete words being grouped in semantic networks, while abstract words are grouped in word association networks (see also Duñabeitia et al., 2009). Therefore, word association might not be just a paradigm through which to study the processing of language but rather a core mechanism taking part in it.

Besides its use as a tool to explore the nature of mental representation of words, word association has also recently shown to be a powerful instrument to computationally estimate lexico-semantic ratings. Traditionally, to obtain such norming, one needed to have a large group of participants to manually score each word one by one (Stadthagen-Gonzalez et al., 2017; Warriner et al., 2013). This process is both time-consuming and expensive, and norming an entire lexicon of a language becomes chimera. However, Van Rensbergen et al. (2016) found that word association provides a way to solve this issue by acting as a proxy for word similarity, which can then be used to extrapolate such ratings without the need of human participants. In their study, they focused on emotional variables, the most relevant being valence – that is, the hedonic value of a word; positive vs. negative – and arousal – that is, its galvanizing value; relaxing vs. activating. Using their data imputation method based on word association, they obtained a 0.91 and 0.84 correlation with the valence and arousal values obtained from human participants.

Yet, despite the evident importance of word association, there is still no clear consensus over the exact mechanisms driving it. One plausible mechanism that has been recurrently discussed as a way for words to become associated is co-occurrence. When two words frequently appear together in texts or speech, they can end up developing an associative relationship. For instance, the cue *stone* commonly elicits *cold* as an associate, although they have no real semantic relationship, because *stone cold* is a common expression, and the same holds for pairs such as *holy* and *cow* or *rock* and *music* (De Deyne et al., 2019)*.* Critically, this is a language-dependent view, since a given expression used in a language may not be used in another (e.g., *stone-cold* do not stand as strong associates in Spanish while *metal-cold* do; Fernández et al., 2018). In this line, several studies have shown that word co-occurrence can be used a measure of word association (e.g., Chaudhari et al., 2010).

This idea, however, puts the question in a chicken-and-egg scenario: Do words become associated because they tend to appear together or do they appear together because they are associated in our mental lexicon? In a recent article, Buades-Sitjar et al. (2021) provided proof for the second option, suggesting that several mechanisms could underlie word association. They based their proposal on the studies by Van Rensbergen et al. (2016), which found that word association data could be used to accurately extrapolate the valence and arousal values of words. Buades-Sitjar et al. suggested that emotionality and concreteness could be driving forces behind word association and that words could become associated due to sharing similar emotional and concreteness values. They proposed that, when conversing about a topic with a certain emotional load and of a specific abstraction level, it is likely that we will need to use words with similar emotional and concreteness values; for instance, when talking about *smile*, speakers may refer to *laughter*, *happiness*, or *fun*. Their study provided proof for this idea by proving that the valence, arousal, and concreteness values of the cue words in word association databases could respectively predict the valence, arousal, and concreteness values of the elicited associates. Moreover, their results were replicable across three different languages – English, Spanish, and Dutch – supporting the notion that this is a core linguistic mechanism regardless of language. Furthermore, these findings were in line with prior observations, showing that the emotional content of short texts can be predicted from the emotional features of words. In this sense, Hsu et al. (2015) correlated affective scores of words and passages from Harry Potter books with brain-activation patterns. Similar emotion-driven activation patterns were found in emotion-related brain areas such as the amygdala and the insula for single words and for passages, respectively. In summary, these results support the notion that word association operates through emotional similarity and concreteness.

However, while Buades-Sitjar et al. (2021) provided a solid starting point of evidence, it alone is not sufficient to fully support their proposal. The association data through which they obtained their results used a classic single-word paradigm in which participants were given a cue word and they had to respond with the first word that came to their minds. While very convenient for laboratory studies, this paradigm is far from being representative of the reality of day-to-day human language, as people do not generally communicate through single, isolated words. Therefore, to support the notion that word association operates through emotional and concreteness similarity, it is critical to find proof that this similarity is also present at more complex levels of language production. Thus, in the present study, we examined whether the emotional and concreteness similarity effect found by Buades-Sitjar et al. in a single-word association paradigm extends to sentence-production paradigms. To this end, we designed a sentence-generation task in which participants were given a cue word and then asked to produce a complete sentence containing said word in a meaningful context. If word association operates through emotional and concreteness similarity, the values of the cue words would be expected to predict the same values of the content words used in the generated sentences. In other words, this study examined the role of emotional dimensions of words (valence and arousal) and concreteness in the lexical associations elicited in a sentence-generation task.

## Methods

### Participants

Thirty-two native Spanish-speaking undergraduate students from Nebrija University voluntarily participated in this study (23 women; average age of 21.9 years; *SD* = 8.56). They were compensated with 10€ for their participation.

### Materials

The experimental material was extracted from Stadthagen-Gonzalez et al.'s (2017) database. In total, 600 words were selected aimed at covering the different valence categories of the database and the largest possible arousal and concreteness spectra. The full list of items together with the resulting sentence-level associates can be found in https://doi.org/10.6084/m9.figshare.17212331.v1 (Duñabeitia, 2021). Of the 600 items, 200 were considered of negative valence (*M* = 2.29; *SD* = 0.44; range = 1.15–3) and 200 other words were classified as neutral (*M* = 5.16; *SD* = 0.26; range = 4.5–5.6). Finally, 200 words of the 600-item list were of positive valence (*M* = 7.69; *SD* = 0.38; range = 7.08–8.7). The mean arousal of the cue words was 5.57 (*SD* = 1.40; range = 1.5–8.45), and the mean concreteness was 4.81 (*SD* = 1.07; range = 1.98–6.73). The average letter length of the cue words was 7.05 letters (*SD* = 2.01; range = 3–14). The average frequency as indicated by the words’ Zipf score was 4.24 (*SD* = 0.64; range = 2.11–6.06).

### Procedure

The experiment was conducted individually in the laboratory. Each participant completed a 50-minute session in which they were presented with a list of words randomly selected from the 600-word pool. In each trial, participants were presented with a fixation cross for 1,000 ms, immediately followed by a cue word that remained on the center of the screen for 3,000 ms. They were then asked to write a sentence where the cue word had to appear in a meaningful context. They were instructed to generate sentences including the cue word, written in a manner that proved that they knew its meaning, and avoiding *proforms* or general-meaning words. They were told not to spend much time in each sentence, as a very relevant aspect of the study consisted of knowing their first impression when they read the cue words given. They typed the sentences using the keyboard and pressed ENTER to move on to the following trial. The words were presented in Spanish, and the sentences produced by the participants were also coded in Spanish language. Stimuli presentation and data collection were done using the Experiment Builder (SR Research, Ontario).

### Data Preprocessing

Given that each participant took a different amount of time to write each sentence, there were between-subject differences in the number of completed trials. The mean number of sentences generated by the participants was 96.25 (*SD* = 21.23; range = 52–147). Sentences that did not include the cue word were eliminated, resulting in a total of 2081 valid sentences. The sentences were then separated into words, and conjunctions, prepositions, determinants, pronouns, and proper nouns (functional words) were removed from the analysis. The aim of this phase consisted of keeping only those words with lexical meaning (nouns, verbs, adverbs, and adjectives), which would be considered as the lexical associates of each cue word. In addition, before analyzing the data, conjugated verbs were changed into their infinitive form and nouns and adjectives were transformed into their singular and masculine form, as that is currently considered the generic form in Spanish. An example of the process would be as follows (translated into English): Given the cue word *dance*, Participant 101 constructed the sentence “Dance is a form of positive body expression.” First, we separated all the words that conformed the sentence, extracted the relevant lexical units, and changed the conjugated verb into its infinitive form (dance|to be|form|positive|body|expression). The last step consisted of analyzing the correspondence of ratings between the cue word given (*dance*) and these words that were considered as its lexical associates. The independent variables were the arousal, valence, and concreteness values of the cue words, while the dependent variables were the values of the same dimensions of the lexical words produced by the subjects in the sentences (the lexical associates; see the Appendix for an extended example). To conclude, the final data set included a total of 12,484 cue–associate pairs. Valence and arousal scores from both cues and associates were extracted from Stadthagen-Gonzalez et al. (2017), and concreteness ratings were extracted from Duchon et al. (2013).

### Results

Piece-wise mixed-model regressions were run to examine whether the emotional and concreteness values of the cue words could predict those of the associates. The models were created using the *lmer* function from the *lme4* package for R (Bates et al., 2015), while the *R*^2^ of the resulting models was computed using the *r.squaredGLMM* function from the MuMIn package for R (Bartoń, 2013). The first step of each analysis included as a factor only the cue value being predicted by the model – that is, when trying to predict the valence of the associate, the valence of the cue was used as a mixed effect. The second step added the other two values of the cue – that is, when trying to predict the valence of the associate, the valence, arousal, and concreteness values of the cue were included in the model. In both instances, participants and items were included as random effects. Importantly, an ANOVA of the two steps of the models showed that including the extra two variables did not significantly improve any of them, and thus, only the first step of each model is reported.

Table 1 displays the results of the linear regressions predicting the valence, arousal, and concreteness values of the associate words of the total 12,484 cue–associate pairs. The model predicting the associates’ valence had an *R*^2^ of .057, and the *B* coefficients were 5.22 for the intercept (*p* < .001) and 0.15 for the cue valence (*p* < .001). The model predicting the associates’ arousal had an *R*^2^ of .028, and the *B* coefficients were 4.59 for the intercept (*p* < .001) and 0.11 for the cue arousal (*p* < .001). The model predicting the associates’ concreteness had an *R*^2^ of .013, and the *B* coefficients were 3.71 for the intercept (*p* < .001) and 0.11 for the cue concreteness (*p* < .001).

**Table 1:** Regressions on the whole set of 12,484 cue–associate pairs

Variable	*B*	*SE*	*t*
Predicted associate valence			
Intercept	5.22	0.04	118.20
Cue valence	0.15	0.01	21.15
Predicted associate arousal			
Intercept	4.59	0.04	106.64
Cue arousal	0.11	0.01	15.58
Predicted associate concreteness			
Intercept	3.71	0.07	50.42
Cue concreteness	0.11	0.01	8.25
*Note*. All values were statistically significant at the *p* < .001 level.			

In a second analysis, we also contrasted the data obtained through our sentence-level task with data obtained from single-word association tasks. To that end, we selected the cue–associate pairs produced by our participants that were also cue–associate pairs in the *Spanish Free-Association Norms* (Fernández et al., 2018), the largest single-word association database in Spanish to date. A total of 1,657 (13.27%) of the cue–associate pairs in our study were also present in the database. The same models applied to the full datasheet were also applied in this instance. Again, the second step of the regression did not significantly improve any of the models. Table 2 displays the results of the linear regressions predicting the valence, arousal, and concreteness values of the associate words. The model predicting the associates’ valence had an *R*^2^ of .25, and the *B* coefficients were 3.59 for the intercept (*p* < .001) and 0.44 for the cue valence (*p* < .001). The model predicting the associates’ arousal had an *R*^2^ of .18, and the *B* coefficients were 3.17 for the intercept (*p* < .001) and 0.40 for the cue arousal (*p* < .001). The model predicting the associates’ concreteness had an *R*^2^ of .01, and the *B* coefficients were 2.92 for the intercept (*p* < .001) and 0.23 for the cue concreteness (*p* < .001).

**Table 2:** Regressions on the 1,657 cue–associate pairs present in Fernández et al. (2018)

Variable	*B*	*SE*	*t*
Predicted associate valence			
Intercept	3.59	0.14	25.29
Cue valence	0.44	0.02	17.50
Predicted associate arousal			
Intercept	3.17	0.16	20.19
Cue arousal	0.40	0.03	14.38
Predicted associate concreteness			
Intercept	2.92	0.30	9.68
Cue concreteness	0.23	0.06	3.97
*Note*. All values were statistically significant at the *p* < .001 level.			

## Discussion

The word association paradigm can be a very useful tool for understanding lexical processing and organization, and it has already provided relevant clues about how words may be interconnected (e.g., Crutch & Jackson, 2011). However, there is still no scientific consensus on the exact mechanisms underlying word association. While there is relative agreement that words are associated by co-occurrence in speech (Chaudhari et al., 2010), recent studies suggest that the association of words is, first and foremost, directed by their similarity in terms of valence, arousal, and concreteness (Buades-Sitjar et al., 2021), since the values of the associates in these dimensions can be effectively predicted from those of the cue words. To verify the idea that words are associated by means of their emotional and concreteness values, we gave participants cue words and asked them to provide a meaningful sentence for each of them. We then analyzed the predictive power that the valence, arousal, and concreteness of cue words had on the words used in the sentences.

The linear regressions revealed that valence, arousal, and concreteness of the cue words were able to predict the same values of these dimensions in their sentence-level associates. Furthermore, when analyzing exclusively the sentence-level associates obtained in this task that also appeared in the single-word association database by Fernández et al. (2018), these results were replicated, in line with those reported by Buades-Sitjar et al. (2021). In addition, these findings align with previous outcomes that showed that the emotional content of small texts can be predicted from the emotional characteristics of their individual component words (Hsu et al., 2015).

In summary, these results support the notion that word association operates through emotional similarity and concreteness. They also indicate that word association extends not only to single-word paradigms but also occurs when a sentence is constructed, providing support for the relevance of textual co-occurrence for word association. Future studies should be aimed at investigating if and how these lexical associations occur in foreign and second languages, and not only in a native one, given the claim of an emotional detachment component in non-native language contexts (e.g., Costa et al., 2014; Iacozza et al., 2017). Finally, to reach deeper conclusions concerning how lexical associations are created, future studies should also assess how the conceptual components of words presenting different degrees of lexical ambiguity modulate lexical co-occurrence. This is especially relevant in the case of polysemous and homonymous words.

Although these results reinforce the predictive capacity of emotional dimensions and concreteness in word associations, they do not necessarily imply that these are the only or major driving forces causing association. However, the similarity in emotion and concreteness between words seems to be a crucial underlying mechanism operating in word association, and it can plausibly respond to an adaptive phylogenetic value, as it can constitute a communicative advantage. Prior evidence has also emphasized the close link that exists between emotion and concreteness. In this line, emotional features seem to play a key role in the representation of abstract concepts (Kousta et al., 2011). Words would be activated together depending on the emotional context and their concreteness level, being more quickly accessible for each specific affective framework. Hence, we suggest that words co-occur in similar emotional and concreteness communicative frameworks and that this would determine their co-occurrence, which would ultimately lead to word association. A word would activate words of similar valence, arousal, and concreteness, which will normally be used in a specific emotional-communicative framework; in turn, those that co-occur more often in a discourse will also develop stronger associations, increasing the probability that they will reappear together.

To conclude, the similarity in valence, arousal, and concreteness of words would serve to predict the values of these same dimensions not only in the case of their associates in single-word paradigms but also in terms of the words produced in sentence contexts. Hence, the present work is a pioneer in highlighting the factors that could associate words at the sentence level, revealing important contributions of valence, arousal, and concreteness as critical mechanisms that underlie lexical interrelation. Thus, the present research expands our psycholinguistic knowledge by highlighting some of the mechanisms that serve to understand and categorize reality and to refer to it, considering that the analysis of the organization of the mental lexicon is a proxy for analyzing how we organize and structure reality through language.

## Notes

**Open Data:** All data and materials for this work can be found at https://doi.org/10.6084/m9.figshare.17212331.v1 (Duñabeitia, 2021). Our manuscript contains no experiment with a completely executed preregistration.

## Appendix

**Table A1:** Examples of correspondence of emotional and concreteness ratings for the cue word “*baile*” (“dance”) and its associates produced by three different participants

Participant	Cue	Valence	Arousal	Concreteness	Associates	Valence	Arousal	Concreteness
101	Baile	7.5	5.95	4.53	Corporal (corporal)	6.15	5.2	
101	Baile	7.5	5.95	4.53	Expression (expression)	6.4	5.25	2.72
101	Baile	7.5	5.95	4.53	Forma (shape)	5.5	4.85	3.11
101	Baile	7.5	5.95	4.53	Positiva (positive)	8	4.45	
101	Baile	7.5	5.95	4.53	Ser (to be)	6.7	4.5	
119	Baile	7.5	5.95	4.53	Aprender (to learn)	7.28	4.85	4.54
119	Baile	7.5	5.95	4.53	Coreografía (choreography)	6.65	6.35	
119	Baile	7.5	5.95	4.53	Encantar (to love)	8.05	5.45	
119	Baile	7.5	5.95	4.53	Nuevo (new)	7.65	5.9	
131	Baile	7.5	5.95	4.53	Curso (course)	5.8	5.85	4.13
131	Baile	7.5	5.95	4.53	Divertido (funny)			
131	Baile	7.5	5.95	4.53	Fin (end)	3.6	5.55	4.54
131	Baile	7.5	5.95	4.53	Loco (crazy)	4.08	6.7	4.26
131	Baile	7.5	5.95	4.53	Ser (to be)	6.7	4.5	
